# LncRNA NEAT1 promotes glioma cancer progression via regulation of miR-98-5p/BZW1

**DOI:** 10.1042/BSR20200767

**Published:** 2021-07-26

**Authors:** Yabin Li, Xirui Wang, Zhihuang Zhao, Jinxing Shang, Gang Li, Ruijian Zhang

**Affiliations:** 1 Third Department of Neurosurgery, Cangzhou Central Hospital, Cangzhou, P.R. China; 2 Department of Neurosurgery, People’s Hospital of Inner Mongolia Autonomous Region, Hohhot, Inner Mongolia, P.R. China

**Keywords:** BZW1, glioma, miR-98-5p, NEAT1

## Abstract

**Background:** Glioma is the most common malignant tumor in the human central nervous system. Long noncoding RNA nuclear paraspeckle assembly transcript 1 (NEAT1) promotes oncogenesis in various tumors. In the present study, we aimed to examine the role of NEAT1 in altering the properties of gliomas.

**Methods:** Quantitative real-time PCR technology was used to determine the expression levels of relevant genes in tumor tissues and cell lines. The protein expression levels were validated by Western blotting. Cell counting kit-8 (CCK-8) and colony formation assays were used to test the cell proliferation ability. A luciferase reporter assay was used to determine the interactions of the genes. Tumor xenografts were used to detect the role of NEAT1 in gliomas *in vivo.*

**Results:** We demonstrated that NEAT1 up-regulated glioma cells and negatively correlated with miR-98-5p in glioma tissues. A potential binding region between NEAT1 and miR-98-5p was confirmed by dual-luciferase assays. NEAT1 knockdown inhibited glioma cell proliferation. The inhibition of miR-98-5p rescued the knockdown of NEAT1 in glioma cells. Basic leucine zipper and W2 domain containing protein 1 (BZW1) was identified as a direct target of miR-98-5p. We also identified that BZW1 was positively correlated with NEAT1 in glioma tissues. NEAT1 knockdown inhibited glioma cell proliferation *in vivo* via miR-98-5p/BZW1.

**Conclusion:** Our results suggest that NEAT1 plays an oncogenic function in glioma progression. Targeting NEAT1/miR-98-5p/BZW1 may be a novel therapeutic treatment approach for glioma patients.

## Introduction

Glioma is the most common malignant tumor that can be found in the central nervous system of humans [1,2]. Despite recent advances in glioma treatment with surgery, radiotherapy and chemotherapy [3–9], the prognosis of patients with malignant glioma remains poor. Therefore, identifying the genes that are critically associated with oncogenesis and determining the function of these genes by further examination will contribute to the discovery of a therapeutic target for glioma. Recently, various studies have shown that noncoding RNAs are involved in glioma carcinogenesis.

LncRNAs are more than 200 nucleotides in length, do not encode any proteins and have been shown to have several biological functions during the progression of cancers, such as transcription, intracellular trafficking, and chromosome remodeling [10,11]. Increasing evidence has shown that lncRNAs have an important function in various human cancers, although the relevant mechanisms remain poorly understood [12–15]. Aberrant overexpression of the long noncoding RNA nuclear-enriched abundant transcript 1 (NEAT1) has been reported in many types of solid tumors, such as lung cancer, esophageal cancer, colorectal cancer, hepatocellular carcinoma and glioma. NEAT1 is one of the most highly up-regulated lncRNAs in recent pangenomic datasets [16–20]. However, there are few studies about the role of nuclear-enriched abundant transcript 1 (NEAT1) in glioma. In our study, we observed that NEAT1 was up-regulated in glioma cells. Further investigations are warranted to elucidate the upstream and downstream mechanisms of NEAT1 overexpression in glioma.

MicroRNAs are a group of small noncoding RNAs (19–25 bp) that are aberrantly expressed in various tumors [21]. MiRNAs are involved in diverse biological processes, such as cell growth, migration, apoptosis and differentiation, by binding to the 3′-UTRs of mRNAs [22,23]. MiRNA-98 (miR-98) is a let-7 family member [24]. Recently, it has been reported that miR-98 inhibits tumor growth by enhancing the antiproliferative effects of vitamin D [25]. Moreover, down-regulation of miR-98 has been associated with the invasive possibility of glioma, and several target genes, such as interleukin 10 (IL-10) and high-mobility group A2 (HMGA2), have been identified [26,27]. However, little is known about the role of miR-98, especially its regulatory mechanism in glioma cancer progression. Currently, our study uncovered that hsa‐miR‐98‐5p was down-regulated in glioma cell lines. By bioinformatics analysis, a negative correlation between NEAT1 and hsa‐miR‐98‐5p was predicted *in vitro*.

The basic leucine zipper and W2 domain containing protein 1 (BZW1) gene, which encodes a 45-kDa protein, contains an N-terminal basic leucine zipper (bZIP) domain for protein–protein interactions and a C-terminal nucleotide (ATP or GTP)-binding domain [28]. Previously, it was reported that BZW1 could activate histone H4 gene transcription via site II, which is required for the cell cycle. Recently, BZW1 was identified as a novel proliferation regulator that promotes the growth of salivary mucoepidermoid carcinoma [29]. However, little is known about the role of BZW1 in glioma. In the present study, we observed that BZW1 was a target gene of hsa‐mir‐98‐5p. The association among NEAT1, hsamir‐98‐5p, and BZW1 remains unknown.

The present study is conducted to investigate the clinical feature about NEAT1 in glioma and identify the molecular mechanism of NEAT1 in the prognosis of glioma. Our current study was designed to explore the underlying mechanisms of NEAT1 function in glioma cells. Overexpression of NEAT1 was able to increase BZW1 expression and hsa‐mir‐98‐5p mimics can inhibit BZW1 via down-regulating NEAT1 levels. NEAT1 may act as a competing endogenous lncRNA to up-regulate BZW1 by sponging hsa‐mir‐98‐5p in glioma. Taken these together, we found that silencing NEAT1 inhibited the proliferation of glioma cells by regulating the miR-98-5p/BZW1 axis. In addition, we validated that the NEAT1/miR-98-5p/BZW1 axis had an oncogenic function in glioma cancer. These findings might indicate a novel therapeutic target in glioma cancer patients.

## Materials and methods

### Clinical specimens

Thirty clinical tumor tissues were collected from surgical resections of brain gliomas performed at the Department of Neurology Cangzhou Central Hospital. The adjacent normal tissues, which are defined as normal in the ‘Results’ section, were obtained 2 cm away from the glioma tissue. All the patients underwent surgery and did not have other therapies. The specimens were snap-frozen in liquid nitrogen and stored at −80°C until used in the present study. This experimental research work has been carried out in accordance with the World Medical Association Declaration of Helsinki. The present study was approved by the Institute Research Ethics Committee of Department of Neurology Cangzhou Central Hospital. Informed consents were signed by all the patients.

### Cell culture

Normal human astrocytes (NHAs) cell line was purchased from BeNA Culture Collection (Beijing, China). The glioma cell lines U87 (ATCC #HTB-14, glioblastoma of unknown origin) was purchased from ATCC (Manassas, VA, U.S.A.). U251 (TCHu58) was purchased from cell bank of the committee, Chinese Academy of Sciences). All the glioma cells were maintained in DMEM (Thermo Fisher Scientific, Waltham, MA, U.S.A.) supplemented with 10% fetal bovine serum (FBS), 100 U/ml penicillin, and 100 μg/ml streptomycin. Cells were grown at 37°C in 5% CO_2_.

### Cell transfection

We cloned the NEAT1 sequence into the pcDNA3.1 vector. The siRNAs of NEAT1, miR-98-5p mimics, and the negative control were all purchased from Guangzhou RiboBio Co., Ltd. (Guangzhou, China). We transfected the vector or the siRNAs using Lipofectamine 2000 reagent (Invitrogen, Carlsbad, CA, U.S.A.) according the protocols provided by the manufacturer.

### Cell proliferation assay and colony formation assay

The cell counting kit-8 (CCK-8) assay (Sigma, St. Louis, MO, U.S.A.) was used to evaluate cell proliferation. Cells were seeded at 1 × 10^3^ cells per well in 96-well plates. Then, CCK-8 was added at different time points. The absorbance was measured at 450 nm using a microplate reader. For the colony formation assays, 1000 cells were seeded in each well of six-well plates and incubated for 2 weeks. The colonies were fixed with 4% paraformaldehyde, stained with Crystal Violet, and counted.

### RNA extraction and quantitative real-time PCR assays

Total RNA was extracted using TRIzol Reagent (Takara, Dalian, China). Then, cDNAs were synthesized using a cDNA Synthesis Reagent Kit (Promega, Madison, Wisconsin, U.S.A.) according to the manufacturer’s instructions. A SYBR Green PCR Master Mix Kit (Roche, Shanghai, China) was used to test the expression levels of relevant genes. GAPDH was used as the cytoplasmic RNA control. U6 was used as the nuclear RNA control. The primers used were as follows: NEAT1 forward, 5′-AATGCTTGTTCCAGAGCCCA-3′; NEAT1 reverse, 5′-AAGAAGGCAGGCAAACAGGT-3′; BZW1 forward, 5′-CTTCCAACCCCTCCATGTGTT-3′; BZW1 reverse, 5′-GGATAGGGGAGGGGAGAGAC-3′; GAPDH forward, 5′-GAAGACGGGCGGAGAGAAAC-3′; GAPDH reverse, 5′-CCATGGTGTCTGAGCGATGT-3′; U6 forward, 5′-CTCGCTTCGGCAGCACA-3′; and U6 reverse, 5′-AACGCTTCACGAATTTGCGT-3′. The relative quantification value for each target gene is expressed as 2^−ΔΔ*C*_t_^.

### Luciferase reporter assay

We predicted the potential miR-98-5p binding sites of NEAT1 using a database (StarBase v2.0). The putative miR-98-5p target binding sequence in NEAT1 and the mutated binding sites were synthesized and cloned into the pGL3-control vector. The target genes of miR-98-5p were predicted using the bioinformatics database TargetScan (http://www.targetscan.org). To examine whether miR-98-5p targets BZW1 directly, the 3′-UTR fragment of the BZW1 gene and its mutated putative miR-98-5p binding site were subcloned into a pGL3-control plasmid to form the reporter vectors BZW1-3′-UTR-wild-type (BZW1-3′UTR-Wt) and BZW1-3′-UTR-mutated-type (BZW1-3′UTR-Mut), respectively. U87 cells were seeded in 96-well plates (Corning, NY, U.S.A.) for 24 h, and cells at 60–80% confluence were cotransfected with the appropriate vector constructed with either wild-type or mutant and the miR-98-5p-overexpression plasmid or miRNA NC plasmid using Lipofectamine 2000 Reagent according to the manufacturer’s instructions. Luciferase activity was measured 48 h after transfection using the Dual-Luciferase Reporter System (Promega, Madison, WI, U.S.A.), and relative firefly luciferase activity was expressed as the ratio of firefly luciferase activity to *Renilla* luciferase activity.

### RNA pulldown assay

Briefly, 3 × 10^6^ U251 cells were seeded into the dish for 24 h. Next, the cells were transfected with biotin-labeled lncRNA NEAT1 at a final concentration of 100 nM. The cells were harvested at 48 h post-transfection. Incubated with activated streptavidin-dyna beads (Dyna beads M-280 Streptavidin, #11205D, Invitrogen) 10 μl per sample yeast tRNA (10 mg/ml stock; Ambion, Austin, U.S.A.) and 10 μl BSA (10 ng/ml stock) in the lysis buffer (480 μl) with rotation at 4°C for 0.5 h. Then beads were washed, and the sample lysates (600 μl) were mixed with pre-coated beads (50 μl per sample) and incubated at 4°C for 4 h on a rotator. The beads were then pelleted down the next day to remove unbound materials at 4°C for 2 min, 500×***g***, and were washed six times with 500 μl of ice cold lysis buffer. The miR-98-5p levels in the pulldown samples were detected by quantitative real-time polymerase chain reaction (qRT-PCR) and normalized using U6 as an internal reference.

### Western blot assay

The cells were washed twice with PBS and lysed with RIPA buffer (Thermo Fisher, Shanghai, China). The samples were centrifuged at 15000 rpm for 15 min at 4°C. Then, the supernatant of each sample was collected for use. Equal amounts of protein were separated by 10% SDS/PAGE and transferred on to polyvinylidene difluoride membranes (Millipore, MA, U.S.A.). The membranes were blocked with 5% skim milk at room temperature for 2 h and then incubated with primary antibodies overnight at 4°C. The antibodies used in the present study were rabbit anti-BZW1 (1:1000, Abcam, Cambridge, U.K.) and mouse anti-GAPDH (1:2000, Abcam, Cambridge, U.K.). The membranes were incubated with the appropriate horseradish peroxidase-conjugated secondary antibodies at room temperature for 2 h. The bound antibodies were detected using an enhanced chemiluminescence detection system (GE Healthcare, Piscataway, NJ, U.S.A.). The optical density of the resulting bands was analyzed using Image software and was normalized to the GAPDH signal.

### RNA FISH assay

We performed RNA FISH with modification of published protocol. Briefly, RNA probe were labeled by biotin and cells were treated with with 0.5% Triton X-100 in CSK (100 mM NaCl, 300 mM sucrose, 3 mM MgCl_2_, 10 mM PIPES, pH 6.8) buffer and fixed with cold 4% paraformaldehyde. Then, slides were stored at 4°C in 70% ethanol. The slides were dehydrated through an ethanol series to 100% ethanol, and hybridized overnight at 37°C. The cells were washed and the biotin signal detected using FITC-conjugated avidin antibodies, and the cell nucleus were stained with DAPI. Images were collected using an OLYMPUS BX51 microscope. All reagents were RNase-free.

### Tumor xenograft animal experiments

We constructed the stable cell lines as described previously. Then, the stable si-NEAT1-overexpressing U87 cells or control cells were inoculated (5 × 10^6^ cells per site) bilaterally on the axillary fossae of female BALB/cA-nu mice (6–8 weeks old). All *in vivo* xenograft experiments were performed in Cangzhou Central Hospital Animal Welfare Center. Mice were placed five per cage with free access to water and food.The tumor size was monitored by measuring the length and width with calipers, and the volumes were calculated with the formula: (length × width^2^)/2. The mice were killed by CO_2_-mediated euthanasia, and the tumors were collected for analysis.

### Statistical analysis

The chi-square test was used to determine whether there was a significant difference between the two groups. The correlation in tissues was examined using two-sided Pearson’s correlation. Statistical significance was determined using ANOVA for functional analysis. Pearson’s correlation coefficient was used to determine the correlation between miR-98-5p and NEAT1 in glioma tissues. All statistical analyses were performed using SPSS 20.0 software and GraphPad Prism 6.0 software. The error bars in the figures represent SD. *P<0.05 and **P<0.01 were regarded as statistically significant.

## Results

### NEAT1 is negatively correlated with miR-98-5p in glioma tissues

**Figure 1:**
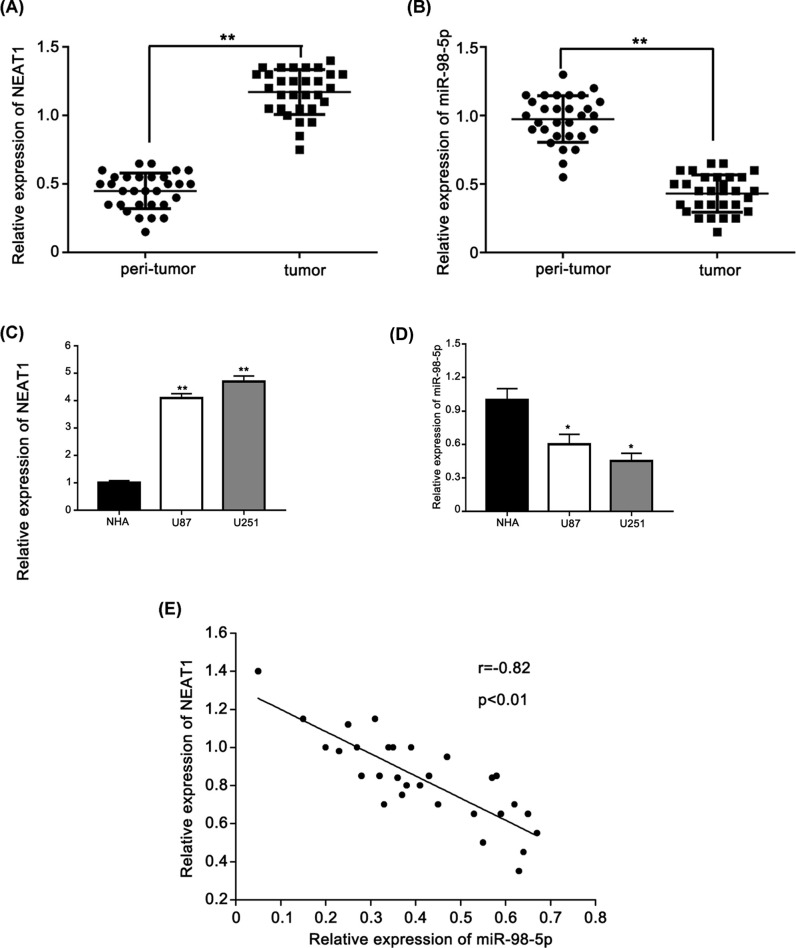
NEAT1 is negatively correlated with miR-98-5p in glioma tissues (**A**) qRT-PCR analysis of the expression of NEAT1 in 30 glioma tissues and peri-tumor tissues. (**B**) qRT-PCR analysis of the expression of miR-98-5p in 30 glioma tissues and peri-tumor tissues. (**C**) qRT-PCR analysis of the expression of NEAT1 in two glioma-derived cell lines and one normal cell line. (**D**) qRT-PCR analysis of the expression of miR-98-5p in two glioma-derived cell lines and one normal cell line. (**E**) The correlation between NEAT1 level and miR-98-5p level was examined by qRT-PCR analysis in 30 cases of glioma tissues. *P<0.05, **P<0.01 as compared with the control. The experiments were run three times.

To identify the role of lncRNA NEAT1 in glioma, we performed real-time PCR to measure the expression level of lncRNA NEAT1 in 30 pairs of glioma tissues. LncRNA NEAT1 was up-regulated in glioma tissues compared with that in peritumor tissues (Figure 1A). Next, we performed real-time PCR to measure the expression of miR-98-5p in 30 pairs of glioma tissues. Our data showed that the expression level of miR-98-5p was down-regulated in glioma tissues compared with that in peritumor tissues (Figure 1B). Furthermore, NEAT1 and miR-98-5p were validated in NHAs cell line and in two glioma cell lines. NEAT1 expression was increased in the glioma cells compared with that in the NHA cells (Figure 1C). In contrast, miR-98-5p expression was down-regulated in the glioma cells compared with that in the NHA cells (Figure 1D). In addition, we found that the expression level of NEAT1 was negatively correlated with the expression of miR-98-5p in glioma cancer tissues (Pearson’s correlation coefficient r = −0.82, *P*<0.01) (Figure 1E). In conclusion, NEAT1 is negatively correlated with miR-98-5p in glioma tissues.

### NEAT1 directly targeted miR-98-5p

**Figure 2:**
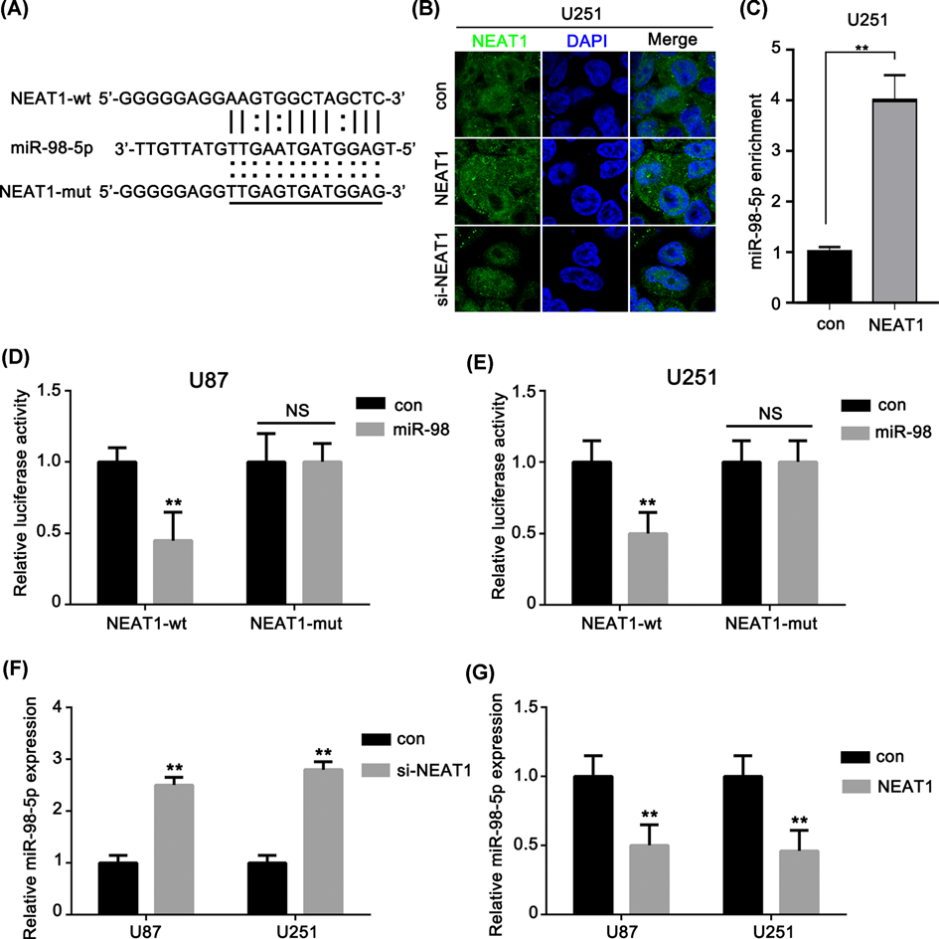
NEAT1 directly targeted miR-98-5p (**A**) Sequences of miR-98-5p and the potential miR-98-5p-binding sites of NEAT1. Seed sequences and mutant are marked. (**B**) RNA-FISH tested NEAT1 in U251 cells by Olympus BX51 microscope, magnification 400×, bar = 10 μm. (**C**) RNA pulldown was performed to enrich miRNAs interacted with lncRNA NEAT1 in U251 cells. (**D,E**) Relative luciferase activity was performed by dual-luciferase reporter assay. (**F,G**) Effect of knockdown of NEAT1 on the expression of miR-98-5p in glioma cells. Relative expression levels of miR-98-5p were detected by qRT-PCR. **P<0.01 as compared with the control. The experiments were run three times.

It has been demonstrated that lncRNAs function as competing endogenous RNAs (ceRNAs) by modulating the expression of miRNAs. We predicted the potential binding sites of miR-98-5p to NEAT1 (Figure 2A) Then, we validated that the NEAT1 located in both nucleus and cytoplasm of U251 cells by RNA-FISH (Figure 2B). In order to further determine the binding relationship between lncRNA NEAT1 and miR-98-5p, we used biotin-labeled lncRNA NEAT1 to carry out RNA pulldown and then used qRT-PCR to analyze miR-98-5p enrichment. The results showed that the abundance of miR-98-5p in biotin-labeled lncRNA NEAT1 group was significantly higher than that in control group (Figure 2C). This suggested that NEAT1 could function as ceRNAs in glioma. To clarify the underlying mechanism, a dual-luciferase reporter assay was performed in U87 and U251 cells. The results showed that the relative luciferase activity was greatly reduced in the group cotransfected with miR-98-5p and NEAT1-wt compared with that in the group cotransfected with pre-NC and NEAT1-wt. On the contrary, the relative luciferase activity was not significantly reduced in cells cotransfected with miR-98-5p and NEAT1-mut (Figure 2D,E). Furthermore, we detected the expression levels of miR-98-5p in the U87 and U251 cells treated with si-NEAT1 or with overexpressed NEAT1, respectively. The expression level of miR-98-5p was increased with si-NEAT1 treatment (Figure 2F). In contrast, the expression level of miR-98-5p was reduced with NEAT1 overexpression (Figure 2G). Overall, these results suggested that NEAT1 directly targeted miR-98-5p.

### Repression of miR-98-5p could reverse si-NEAT1-induced glioma cell tumorigenesis inhibition

**Figure 3:**
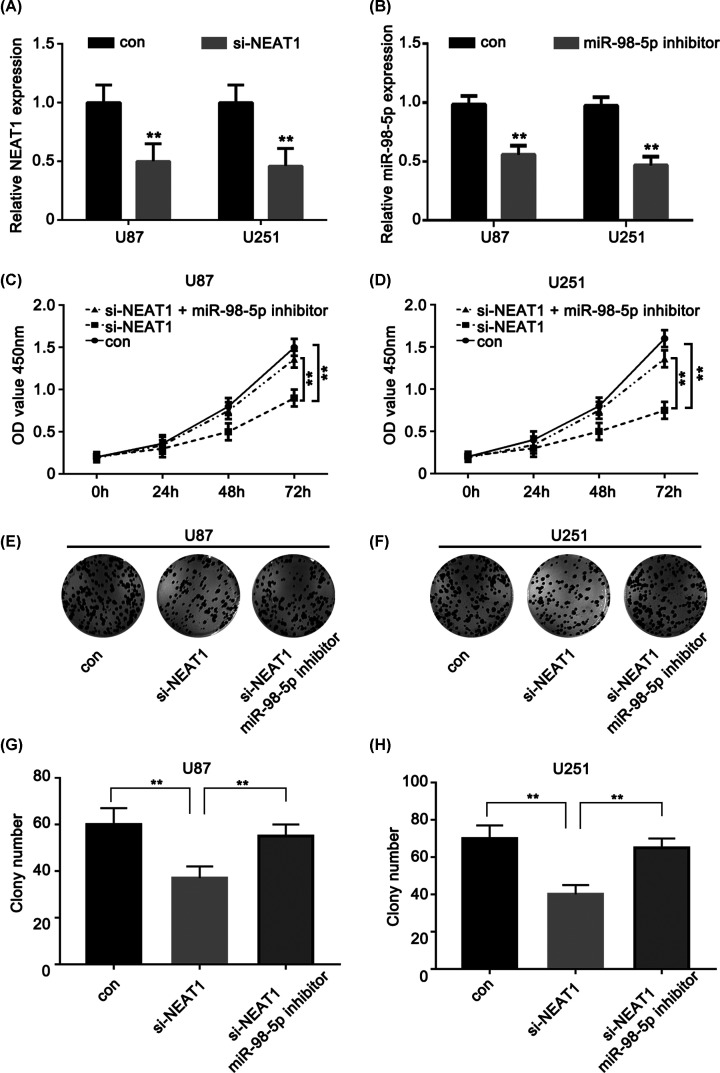
Repression of miR-98-5p could reverse the si-NEAT1-induced glioma cell tumorigenesis inhibition (**A**) NEAT1 expression levels were evaluated using qRT-PCR in si-NEAT1-transfected U87 and U251 cells. (**B**) miR-98-5p expression levels were evaluated using qRT-PCR in miR-98-5p inhibitor transfected U87 and U251 cells. (**C,D**) CCK-8 assay was performed to determine the proliferation of U87 and U251 cell lines with different treatment. (**E,F**) Colony formation assays the U87 and U251 cell lines with different treatment. (**G,H**) Colony number of the U87 and U251 cell lines with different treatment. **P<0.01 as compared with the control. The experiments were run three times.

To assess the potential functional role of NEAT and miR-98-5p, NEAT1-siRNA and an miR-98-5p inhibitor were transfected and the transfection efficiency was measured in the U87 and U251 cell lines, respectively (Figure 3A). Next, we validated the function of miR-98-5p and NEAT1 in the U87 and U251 cells using a CCK-8 kit. The knockdown of NEAT1 resulted in significantly decreased proliferation of the U87 and U251 glioma cells compared with that of cells in the respective control group; the miR-98-5p inhibitor rescued the change in si-NEAT1 expression, resulting in tumor reduction (Figure 3C,D). Moreover, colony formation analysis showed the same event as above (Figure 3E–H). Taken together, these data suggest that the repression of miR-98-5p could reverse si-NEAT1-induced glioma cell tumorigenesis inhibition.

### miR-98-5p directly targets the oncogene BZW1

**Figure 4:**
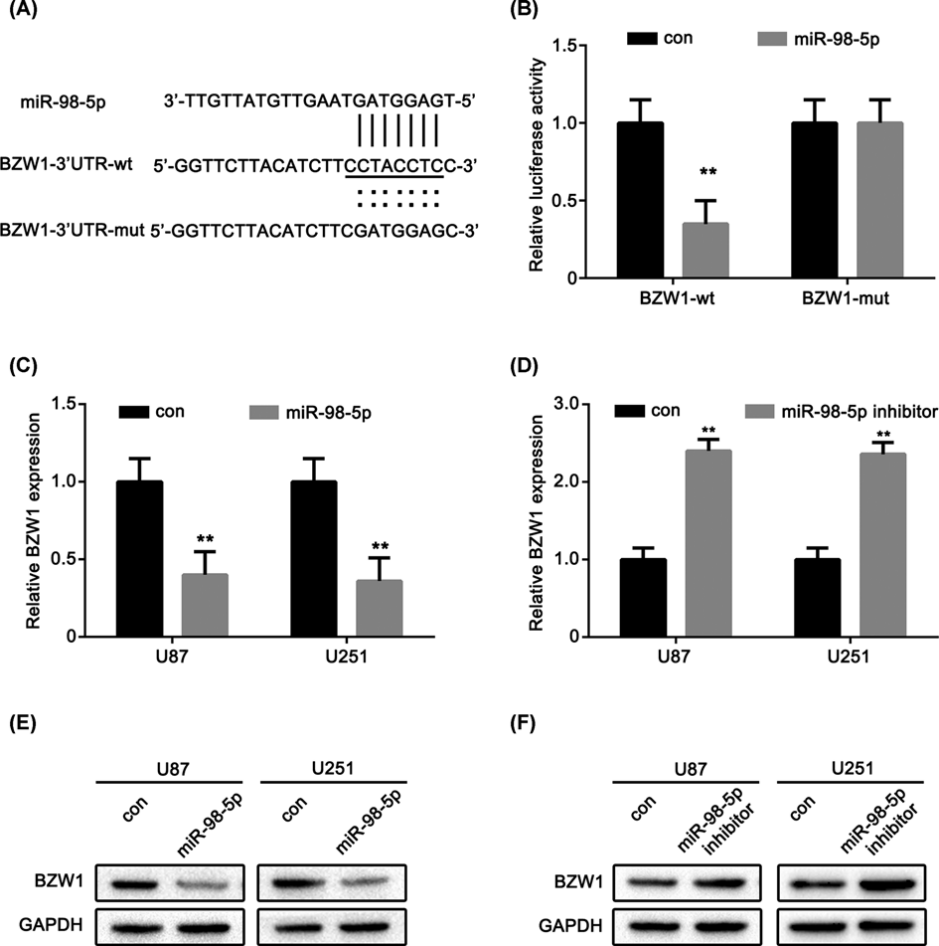
miR-98-5p directly targets oncogene BZW1 (**A**) Sequences of miR-98-5p and the potential miR-98-5p-binding sites of BZW1. Seed sequences and mutant are marked. (**B**) Relative luciferase activity was performed by dual-luciferase reporter assay in U87 cells. (**C,D**) BZW1 expression levels were evaluated using qRT-PCR in miR-98-5p overexpression or miR-98-5p inhibitor transfected U87 and U251 cells, respectively. (**E,F**) BZW1 expression levels were evaluated using Western blot in miR-98-5p overexpression or miR-98-5p inhibitor transfected U87 and U251 cells, respectively. **P<0.01 as compared with the control. The experiments were run three times.

We used TargetScan, PicTar, and miRanda software to predict the potential targets of miR-98-5p. As a result, the top 100 potential targets were identified. Among these genes, BZW1 was up-regulated. We hypothesized that BZW1 may be a direct target of miR-98-5p in glioma. The putative binding site of the microRNA in the 3′-UTR of BZW1 and the complementary seed sequences were mutated (Figure 4A). Next, a luciferase assay was performed to determine whether BZW1 was a direct target of miR-98-5p in glioma cells. The luciferase activity generated by the reporter vector with BZW1-3′UTR-wt decreased after co-transfection with miR-98-5p when compared with that in the control group. However, the luciferase activity generated by the reporter vector with BZW1-3′UTR-mut had no change compared with that in the control group (Figure 4B). We detected the effects of miR-98-5p overexpression and inhibition on the expression of BZW1 using real-time PCR technology. The results showed that miR-98-5p overexpression decreased the protein expression of BZW1 (Figure 4C), whereas miR-98-5p inhibition had the opposite effect (Figure 4D). The following Western blot assays showed that the relative protein level of BZW1 in the U87 and U251 cells was significantly decreased after transfection with miR-98-5p, while blockage of miR-98-5p could increase the BZW1 expression level in glioma cells (Figure 4E,F). These results indicated that miR-98-5p might suppress the expression of BZW1 by strongly and directly binding to the putative site in its 3′-UTR.

### BZW1 is positively correlated with NEAT1 in glioma

**Figure 5:**
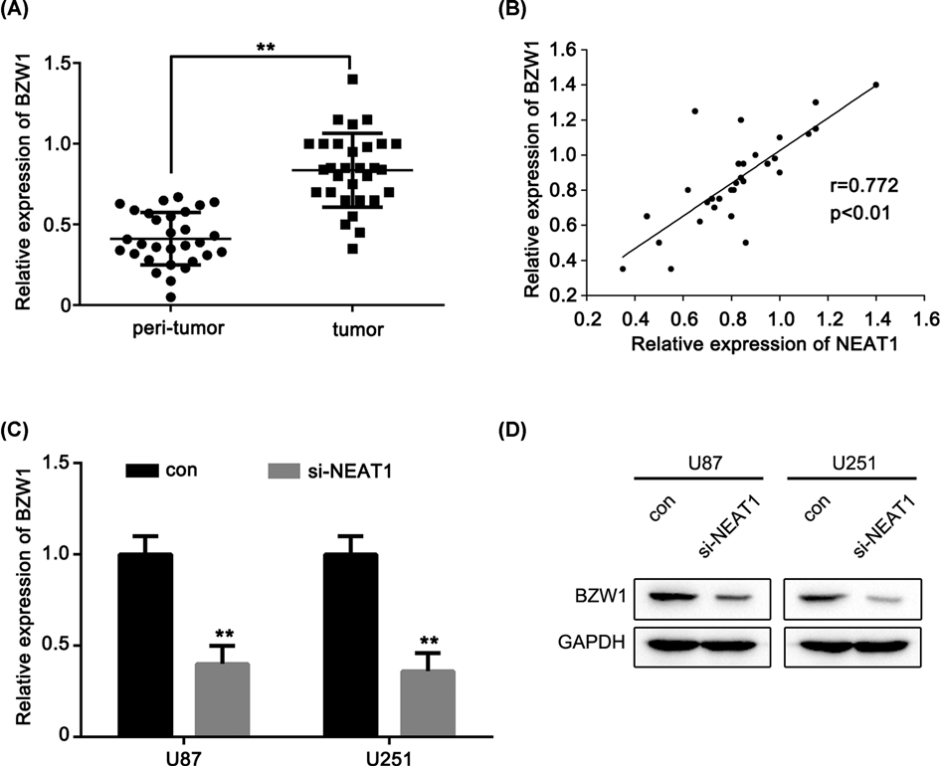
BZW1 is positively correlated with NEAT1 in glioma (**A**) qRT-PCR analysis of the expression of BZW1 in 30 glioma tissues and peri-tumor tissues. (**B**) The correlation between NEAT1 level and BZW1 level was examined by qRT-PCR analysis in 30 cases of glioma tissues. (**C**) BZW1 expression levels were evaluated using qRT-PCR in si-NEAT1 transfected U87 and U251 cells, respectively. (**D**) BZW1 expression levels were evaluated using Western blot in si-NEAT1 transfected U87 and U251 cells, respectively. **P<0.01 as compared with the control. The experiments were run three times.

Having confirmed that NEAT1 could regulate miR-98-5p expression and that miR-98-5p directly targets BZW1, whether NEAT1 exerts its oncogenic activity though the miR-98-5p/BZW1 axis remains unclear. We validated the expression level of BZW1 in glioma tissues using real-time PCR. The results showed that BZW1 was up-regulated in glioma tissues compared with that in peritumor tissues (Figure 5A). We found that the expression level of NEAT1 was positively correlated with the expression of BZW1 in glioma cancer tissues (Figure 5B). Moreover, the inhibition of NEAT1 largely suppressed the mRNA and protein expression levels of BZW1 in the U87 and U251 cells by si-NEAT1 (Figure 5C,D). These results strongly suggested that BZW1 is positively correlated with NEAT1 in glioma.

### BZW1 rescues the si-NEAT1-induced glioma cell tumorigenesis inhibition

**Figure 6:**
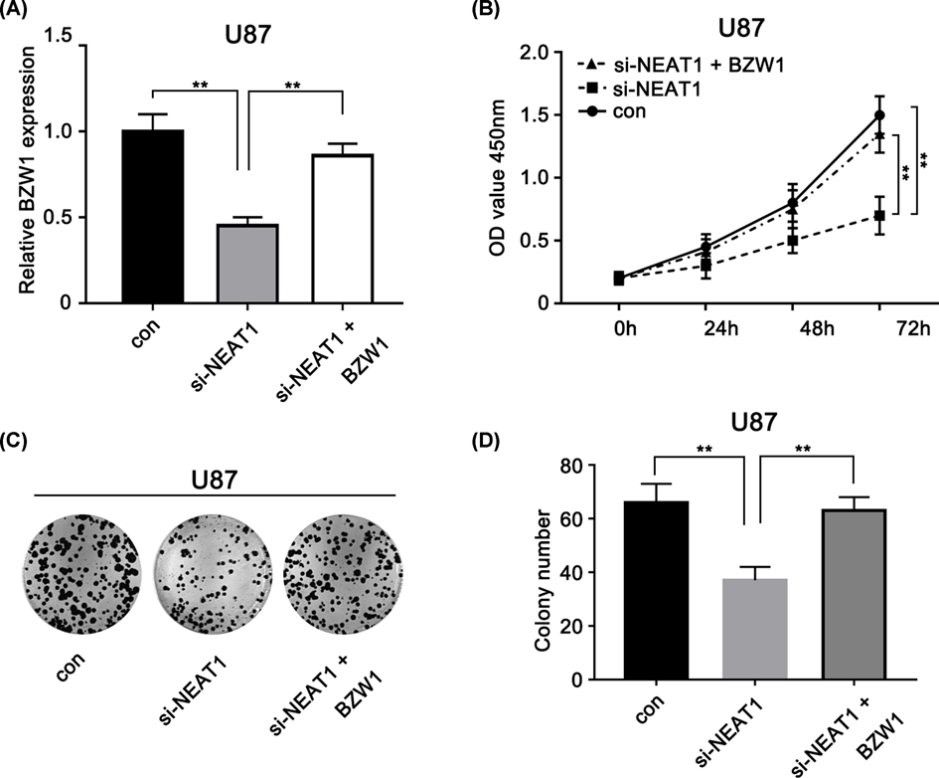
BZW1 rescues the si-NEAT1-induced glioma cell tumorigenesis inhibition (**A**) BZW1 expression levels were evaluated using qRT-PCR in si-NEAT1 or both si-NEAT1and BZW1-transfected U87 cells. (**B**) CCK-8 assay was performed to determine the proliferation of U87 cell lines with different treatment. (**C,D**) Colony formation assays the U87 cell lines with different treatment. **P<0.01 as compared with the control. The experiments were run three times.

In order to validate the role of BZW1 in NEAT1/miR-98 axis in glioma, NEAT1-siRNA and BZW1 vector were transfected and the transfection efficiency was measured in the U87 (Figure 6A). Next, we validated the function of BZW1 and NEAT1 in the U87 cells using a CCK-8 kit. The knockdown of NEAT1 resulted in significantly decreased proliferation of the U87 glioma cells compared with that of cells in the respective control group; the BZW1 overexpression rescued the change in si-NEAT1 expression, resulting in tumor reduction (Figure 6B). Moreover, colony formation analysis showed the same event as above (Figure 6C,D). Taken together, these data suggest that the overexpression of BZW1 could rescue si-NEAT1-induced glioma cell tumorigenesis inhibition and that NEAT1’s oncogenic activity occurs partly via the miRNA-98-5p/BZW1 axis in glioma cells.

### Knockdown of NEAT1 inhibits glioma cell growth *in vivo*

**Figure 7:**
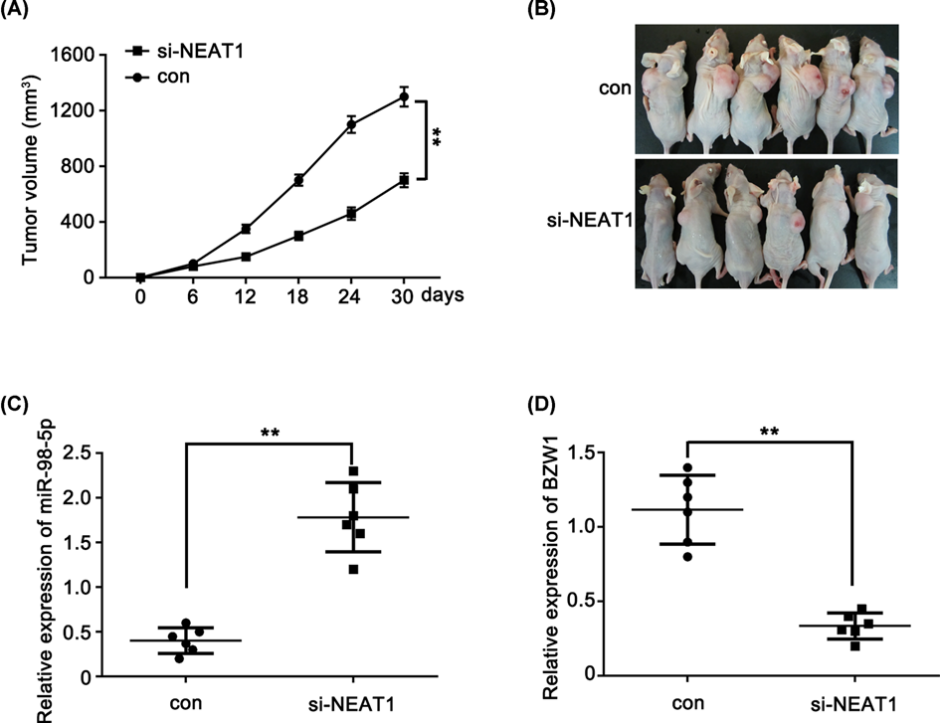
Knockdown of NEAT1 inhibits glioma cell growth *in vivo* (**A**) Tumor growth curves of subcutaneous implantation models of U87 cell are shown. (**B**) The tumor derived from stable overexpression of siNEAT1-U87 cells was smaller than that of control group (*n*=6 in each group). (**C**) Real-time PCR results demonstrated that the expression of miR-98-5p was higher in the si-NEAT1 induced tumors compared with the control group. (**D**) qRT-PCR results demonstrated that the expression of BZW1 was lower in the si-NEAT1 induced tumors compared with the control group. **P<0.01 as compared with the control.

A stable si-NEAT1 knockdown U87 cell line and a control cell line were established to confirm that the oncogenic activity of NEAT1 was partially mediated via regulation of the miRNA-98-5p/BZW1 axis *in vivo*. The cell lines were injected into the axillary fossa of SCID mice, and the tumor growth activity was measured. The average volume of the tumors derived from the si-NEAT1 group was only 35% of that in the control group (Figure 7A,B). Furthermore, we measured the expression levels of miR-98-5p and BZW1 in two groups of tumors. The results revealed higher expression of miR-98-5p (Figure 7C) and lower expression of BZW1 (Figure 7D) in the si-NEAT1 group than in the control group. These results were consistent with the effects of NEAT1 knockdown *in vitro.* These findings strongly suggest that NEAT1 regulates glioma cell proliferation via the miR-98-5p/BZW1 axis.

## Discussion

Glioma is a highly fatal disease in the human central nervous system and is a major contributor to death in patients with tumors. Thus, novel therapeutic approaches targeting glioma are urgently needed. Growing evidence strongly indicates that aberrant lncRNA expression affects biological processes in tumors as well as regulation of the cell cycle, differentiation, and apoptosis in normal cells [30,31]. For instance, the functions of many lncRNAs, such as HOTAIR, H19 and GAS5, have been revealed [32–34]. NEAT1 has earned a reputation as a transcriptional regulator for numerous genes. Moreover, NEAT1 has been found to be correlated with poor survival in patients with various cancers [35–38]. In this study, we evaluated that NEAT1 expression levels in glioma tissues were higher than those in peritumor tissues. Studies indicated that lncRNAs play an important role in multiple process in cells acting as molecular sponges for the regulation of microRNAs. For instance, lncRNA GAS5 and CCAT2 have been indicated as sponges in promoting cancer development [39–41]. Moreover, hsa‐mir‐98‐5p was down-regulated in glioma tissues than those in peritumor tissues. Inhibition of NEAT1 can reduce the promote progression and hsa‐mir‐98‐5p mimics were able to reverse this process. We found that NEAT1 levels in 30 human glioma tissues were higher than those in peritumor tissues, which is consistent with the results of previous studies. Besides this, we also found that BZW1 is a target gene of hsa‐mir‐98‐5p. Therefore, we hypothesized that NEAT1/hsa‐mir‐98‐5p/BZW1 axis was involved in the progression of glioma cancer.

NEAT1, serving as a significant cancer‐related lncRNA, can lead to several serious cancers [36,37,42–44]. In bladder cancer, NEAT1 has played oncogenic roles and has been indicated as a therapeutic target [45]. NEAT1 was able to promote NSCLC carcinogenesis through activating the miR‐377‐3p/E2F3 pathway [46]. In gastric cancer, NEAT1 serves as a bad prognostic factor and can promote cancer growth [36]. Besides these, it is reported that by regulating miRNA‐214 NEAT1 can promote malignant thyroid carcinoma [47]. By activating microRNA let‐7e, down-regulation of NEAT1 can repress the advanced progression of glioma stem cells [48]. miRNA-449a is able to inhibit cell growth in lung cancer and mediate NEAT1 [49]. Therefore, the interaction between lncRNAs and microRNAs plays an important role in carcinogenesis. Our results manifested that the expression of NEAT1 and hsa-mir‐98‐5p was showing a significant negative correlation in glioma cancer. NEAT1 down-regulation distinguishingly up-regulated the expression of hsa‐mir‐98‐5p and it was demonstrated that hsa‐mir‐98‐5p could reverse the effects of NEAT1 on glioma progression. Some of previous researches’ work showed that NEAT1 participated in glioma development. Such as Zhen et al. found that knockdown of NEAT1 repressed the malignant progression of glioma through sponging miR‐107 and inhibiting CDK14 [50]. Moreover, lncRNA NEAT1 promotes glioma pathogenesis though many of other pathways including NEAT1/miR-449b-5p/c-Met axis, NEAT1/MiR-92b, TLR9/NEAT1/STAT3, NEAT1/microRNA let-7e, miR-152-3p/CCT6A, and miR-139-5p/CDK6 [51–56]. In our present study, we further demonstrated that NEAT1 was up-regulated and acted as ceRNA in gliomas. This result coincides with the work of predecessors.

It has been reported that the alteration of miRNA in carcinoma and non-carcinoma cells has great molecular and clinical implications. We focused on determining which miRNA was affected by NEAT1 in glioma. Finally, we discovered miR-98-5p by bioinformatics analysis. miR-98-5p has been reported in several types of cancers. Deregulations of miR‐98-5p lead to the development and progression of some cancers [57–62]; for instance, some studies have reported that miR‐98 exhibits suppressive effects on glioma and melanoma [63,64]. However, reversely, miR‐98 has been found to be significantly up-regulated in various cancers such as gastric cancer, colon cancer, and small cell lung cancer [65–67]. Hence, the miR‐98 may exhibit antitumor or oncogenic functions in different cancer. In the present study, we found that the hsa‐mir‐98‐5p levels were significantly decreased in both glioma tissues and cells compared with control group. Moreover, hsa‐mir‐98‐5p mimics may reverse the siNEAT1 effects in glioma cells. Recently, the interactions between lncRNAs and miRNAs have attracted a lot of attention. We further focused on how NEAT1 and hsa‐mir‐98‐5p interact with each other. In our study, we found that miR-98-5p was negatively correlated with NEAT1 and was a target of NEAT1. Bioinformatics analyses verified that miR-98-5p could bind to NEAT1 directly. In addition, luciferase reporter genes and real-time PCR assays showed that miR-98-5p directly bound to NEAT1. Next, knockdown of NEAT1 significantly increased the expression of miR-98-5p in glioma cells and overexpression of NEAT1 significantly induced the expression of miR-98-5p in glioma cells.

The basic leucine zipper and W2 domains 1 (BZW1) is a member of the bZIP superfamily of transcription factors. The BZW1 gene encodes a 45-kDa protein containing an N-terminal bZIP domain for protein–protein interaction and a C-terminal nucleotide (ATP or GTP) binding domain [11]. Human BZW1 can activate histone H4 gene transcription and serves as a co-regulator of other transcription factors for the control of cell cycling [68]. There are few reports have reported that BZW1 is involved in cancer. In our study, we first reported that BZW1 is target gene of has-mir-98-5p by using bioinformatics analysis. We found that mir-98-5p directly targeted the 3′UTR of BZW1 mRNA through luciferase report gene analysis. Then, we validated that BZW1 was a direct target of miR-98-5p using real-time PCR and Western blot analyses. Knockdown of NEAT1 was able to decrease the expression of BZW1 while up-regulating miR-98-5p, which can inhibit glioma progression *in vivo*. These results suggest that NEAT1 promotes glioma pathogenesis at least partly by regulating the miR-98-5p/BZW1 axis.

Finally, we investigated whether NEAT1 regulated miR-98-5p expression. Our data suggest that NEAT1 could promote glioma tumorigenesis via the miR-98-5p/BZW1 axis *in vivo*.

In summary, this may be the first discovery that the NEAT1/ miR-98-5p/BZW1 axis plays an important role in glioma. Our findings indicate that this axis may represent a novel prognostic indicator and a target for gene therapy in glioma.

## Conclusion

LncRNA NEAT1 promotes glioma cancer progression via regulation of the miR-98-5p/BZW1 pathway. The inhibition of lncRNA NEAT1 or BZW1 might be an effective therapeutic strategy to treat glioma cancers.

## Data Availability

All data associated with the present study are included in this manuscript. All the data are available from the corresponding author Yabin Li (liyb01@hotmail.com) on reasonable request.

## Abbreviations

bZIP: basic leucine zipper
BZW1: basic leucine zipper and W2 domain containing protein 1
CCK-8: cell counting kit-8
ceRNA: competing endogenous RNA
E2F3: E2F transcription factor 3
FISH: fluorescence in situ hybridization
GAPDH: glyceraldehyde-3-phosphate dehydrogenase
NEAT1: long noncoding RNA nuclear-enriched abundant transcript 1
NHA: normal human astrocyte
qRT-PCR: quantitative real-time polymerase chain reaction
SCID: severe combined immune-deficient
U6: U6 small nuclear RNA
